# Regulated Monosyllabic Talk Test vs. Counting Talk Test During Incremental Cardiorespiratory Exercise: Determining the Implications of the Utterance Rate on Exercise Intensity Estimation

**DOI:** 10.3389/fphys.2022.832647

**Published:** 2022-03-30

**Authors:** Siti Ruzita Mahmod, Leela T. Narayanan, Rumaisa Abu Hasan, Eko Supriyanto

**Affiliations:** ^1^Cardiorespiratory Physiotherapy Laboratory, School of Biomedical Engineering and Health Sciences, Faculty of Engineering, Universiti Teknologi Malaysia, Johor Bahru, Malaysia; ^2^Department of Biomedical Engineering, School of Biomedical Engineering and Health Sciences, Faculty of Engineering, Universiti Teknologi Malaysia, Johor Bahru, Malaysia

**Keywords:** cardiorespiratory, exercise intensity, talk test, heart rate, monitoring, prescription

## Abstract

**Purpose:**

When utilizing breathing for speech, the rate and volume of inhalation, as well as the rate of exhalation during the utterance, seem to be largely governed by the speech-controlling system and its requirements with respect to phrasing, loudness, and articulation. However, since the Talk Test represents a non-standardized form of assessment of exercise intensity estimation, this study aimed to compare the utterance rate and the estimated exercise intensity using a newly introduced time-controlled monosyllabic Talk Test (tMTT) versus a self-paced Counting Talk Test (CTT) across incremental exercise stages and examined their associations with the exercise physiological measures.

**Methods:**

Twenty-four participants, 10 males and 14 females (25 ± 4.0 yr; 160 ± 10 cm; 62 ± 14.5 kg) performed two sessions of submaximal cardiorespiratory exercise at incremental heart rate reserve (HRR) stages ranging from 40 to 85% of HRR: one session was performed with a currently available CTT that was affixed to a wall in front of the participants, and the other session was conducted with a tMTT with a 1-s inter-stimulus interval that was displayed from a tablet. In each session, the participants performed six stages of exercise at 40, 50, 60, 70, 80, and 85% HRR on a treadmill and were also asked to rate their perceived exertion based on Borg’s 6 to 20 Rating of Perceived Exertion (RPE) at each exercise stage.

**Results:**

The newly designed tMTT significantly delineated all the six stages of incremental exercise (*p* ≤ 0.017), while CTT could only delineate exercise stages at 60, 80, and 85% HRR. However, in estimations of exercise intensity, the tMTT demonstrated only moderate associations with HRR and Borg’s RPE, similarly to the CTT.

**Conclusion:**

If the purpose of exercise monitoring is to detect the intensity of light, moderate, and vigorous exercise intensity, the tMTT could be more universally applicable. However, due to its larger variability of speech rate across exercise intensities, the time-regulated approach may alter the speech breathing characteristics of the exercising individuals in other ways that should be investigated in future research.

## Introduction

Vocalization or speaking while exercising at one’s volition results in competition between the breathing patterns required for linguistic phrasing and the exercising muscles (Baker et al., 2008). Consequently, the ability to vocalize comfortably is compromised when cardiorespiratory exercise intensity exceeds a ventilatory threshold (Quinn and Coons, 2011; Rodríguez-Marroyo et al., 2013). Based on this underlying physiological mechanism, various versions of the “speak while exercise” test, also known as the “Talk Test” (TT), have been introduced as tools to gauge exercise intensity. The evaluation approaches in these tests involve measurement of an individual’s reported speaking comfort upon uttering standard text passages at several stages of progressive incremental exercise (Zanettini et al., 2013) as well as calculation of the ratio of the successive counts uttered in a single breath during exercise and those at rest (Norman et al., 2008; Loose et al., 2012).

Utterance production has been found to be strongly associated with pulmonary ventilation and oxygen consumption (Meckel et al., 2002). Moreover, metabolic needs have been shown to significantly outweigh linguistic phrasing at exercise intensities corresponding to 75% of maximal oxygen consumption (Baker et al., 2008) since a high level of ventilatory control is necessary for reasonably normal utterance at higher exercise intensities Hoit et al. (2007). As such, utterance production variables cannot be overlooked in tests that involve vocalization, such as TT, since temporal utterance structure tends to vary even in highly fluent speakers (Zellner, 1994) and may have some effect on outcomes. Additionally, one study investigating the effects of speech production and physiological responses to exercise found that a pre-determined utterance rate of 60–70 words/minute during exercising resulted in a consistent reduction of minute ventilation as exercise intensity increased (Meckel et al., 2002). However, to date, the utterance rate or other utterance characteristics have not been evaluated in any form of TT.

The study aims (1) to compare the utterance rate and the estimated exercise intensity using a newly introduced Talk Test (tMTT) versus a self-paced Counting Talk Test (CTT) across incremental exercise stages and (2) to examine the association between the estimated exercise intensity from the respective TTs and the exercise physiological measures.

## Materials and Methods

### Participants

We included healthy men and non-pregnant women who met the following criteria: the ability to ambulate on a treadmill and no history of cardiovascular disease, significant pulmonary disease, or unstable metabolic disorders. Sample size calculation for correlations was conducted based on a recommended equation (Moinester and Gottfried, 2014). With the number of participants, *n* = 25, and moderate effect size, *d* = 0.5 for bivariate correlation (Sullivan and Feinn, 2012), the statistical power, calculated from the *post hoc* power analysis (Faul et al., 2007) was observed at 0.84. However, only 24 participants (mean age ± standard deviation = 25 ± 4 yr; 10 males) among the university population completed the procedures in this study method and with that number, the statistical power remains the same. The participants were all non-native English speakers and had no reported history of respiratory, speech, or hearing problems. Participants were screened with a Physical Activity Readiness Questionnaire (PAR-Q) as recommended by the American College of Sports Medicine (ACSM). Those who answered “no” to all 7 questions in the PAR-Q were eligible for the exercise testing, and informed consent was obtained from each individual before participation. Participant baseline characteristics are listed in Table 1. The study was approved by the Medical Research Ethics Committee, Ministry of Health Malaysia regarding research with humans (NMRR-15-1614-27729).

**TABLE 1:** Baseline characteristics of the participants.

Characteristics	Values
Age (years)	25 ± 4
Gender	
Male (number, %)	10, 42%
Female (number, %)	14, 58%
Weight (kg) (mean ± SD)	62 ± 14.5
Height (m)	1.6 ± 0.1
Body mass index (kgm^–2^) (mean ± SD)	22.8 ± 3.9
Resting respiratory rate (breaths min^–1^)	
First exercise session (mean ± SD)	18 ± 5
Second exercise session (mean ± SD)	18 ± 5
Resting heart rate (beats min^–1^)	
First exercise session (mean ± SD)	73 ± 8
Second exercise session (mean ± SD)	72 ± 9
Systolic/diastolic blood pressure (mmHg)	
First exercise session (mean ± SD)	110/71 ± 12/7
Second exercise session (mean ± SD)	109/72 ± 12/7
Estimated cardiorespiratory fitness (METs)	
Maximal METs (mean ± SD)	11.5 ± 2.2

### Design

A cross-sectional study was conducted to compare the utterance rate and estimated exercise intensity across different stages of incremental cardiorespiratory exercise that were conducted in two sessions on two different days within 1 week in the Cardiorespiratory Physiotherapy Laboratory, Universiti Teknologi Malaysia. During the first exercise test session, the participants were required to perform the CTT while exercising on a treadmill (Track Motion; Germany) at incremental heart rate reserve (HRR) stages ranging from 40 to 85% of HRR (Loose et al., 2012). In the second session, an identical exercise protocol was repeated with the tMTT instead of the CTT. The treadmill exercise took about 20 to 30 min only from the whole length of the experiment duration, and the participants were informed earlier to refrain from eating at least 2 h before attending the exercise session.

### Methods

During each exercise session, the participants were initially asked to rest in a half-lying position on an adjustable couch while a 12-torso positioned lead electrocardiograph electrodes (Jowett et al., 2005), a pulse oximetry device, and a blood pressure cuff were attached to them. Baseline measurements of the participants’ heart rate (HRrest), respiratory rate, oxygen saturation, and blood pressure were obtained after 5 min of rest. Then, each participant’s maximal heart rate (HRmax) was estimated using the age-predicted maximum heart rate in the HRR equation (208 –0.7 × age) (Tanaka et al., 2001), while the targeted heart rates during the exercise stages at 40, 50, 60, 70, 80, and 85% HRR were calculated using the following equation: [HRR (beats min^–1^) = exercise stages (%) × (HRmax – HRrest) + HRrest] (Cunha et al., 2011).

Next, the participants were equipped with a wireless headset microphone (H8030; Rapoo, Shenzen) and a safety clip on the treadmill before the exercise. The participants could request to stop the exercise test if they experienced discomfort or felt that they could not pursue the test safely, particularly among participants who experienced any deteriorating signs or symptoms such as decreased oxygen saturation less than 85%, persistent irregular heartbeat, muscle cramps, or dizziness. Meanwhile, the standardized instructions for Borg RPE category scale 6 to 20 and the CTT were reviewed for the participants through their headphones to confirm their understanding of the instructions. The instructions and protocols for Borg RPE category scale 6 to 20 and CTT were adopted from Loose et al. (2012), while for tMTT, the standardized instructions were as follows: *“Breath out fully through your mouth. Then, take a deep breath in as much as you can and say out loud at your usual conversational loudness, the following alphabets: | a|, | b|, | c|, | d|, and so on until | z| except | w|. Try to read as many of these alphabets as they appear before having to take another breath. Do not hold your breath when performing this test.”* The Borg 6 to 20 RPE chart and the CTT transcript were affixed to a wall in front of the participants, approximately 90 cm away, while the tMTT during the second exercise session was displayed on a seven-inch tablet affixed on the treadmill front rail.

**FIGURE 1:**
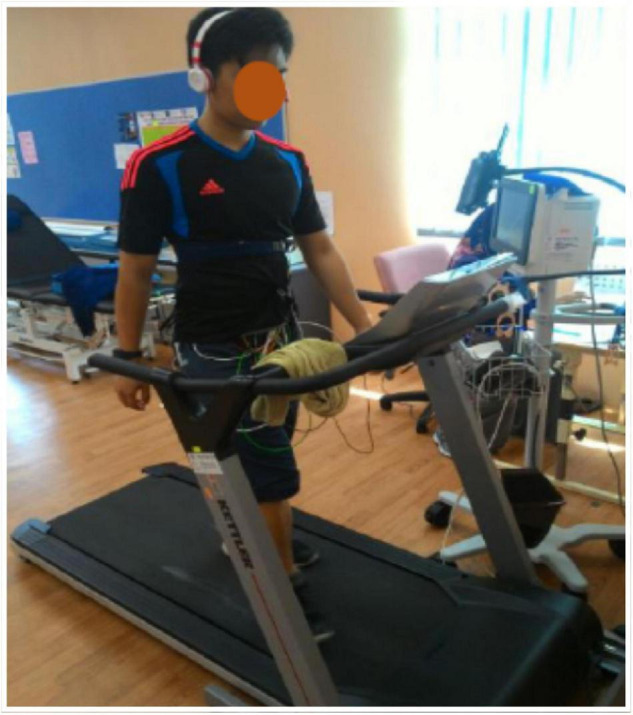
A participant performs an incremental exercise on a treadmill until reaching the targeted heart rates at a predetermined percentage of the heart rate reserve.

**FIGURE 2:**
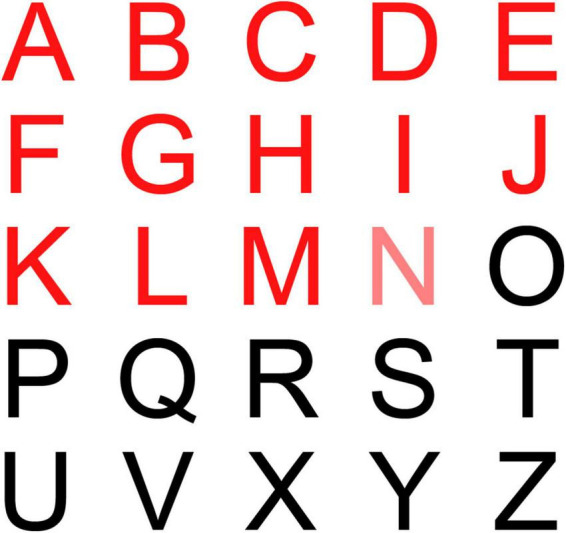
Tablet display of alphabets in the tMTT in front of the participants while they exercised on a treadmill.

Each participant started walking on the treadmill at 3–4 km/h and 0% elevation for 3 min as a warm-up phase. Then, the treadmill grades were increased by adjusting the speed within a range of 1–4 km/h depending on the participants’ tolerance level, and the gradient was increased in 1% intervals until the steady-state targeted heart rates corresponding to 40, 50, 60, 70, 80, and 85% of the HRR were reached (Figure 1). As they reached each stage of the exercise, the participants were asked to rate their perceived exertion based on Borg’s 6 to 20 RPE scale, while they maintained speed and gradient throughout 2-min bouts of each exercise stage. At the last minute of each exercise stage, they were asked to perform the CTT or tMTT immediately by cueing them with the instruction, “take a deep breath in now and start counting or uttering.” During the tMTT, the participants were required to consecutively say out loud as many of the alphabet sounds as possible in a single deep breath and following a pre-set interval of 1 s between the alphabets display. The alphabet that was timed to be uttered was visually cued with font color changes from black into red (Figure 2). They had to control their breath by avoiding any attempt to inhale between alphabet phonation before producing speech expirations to the maximum alphabet phonation they were capable of. They were asked to stop their utterances before taking a second breath. Meanwhile, the participants’ respiratory rate, oxygen saturation, heart rate, and rhythm were monitored throughout the exercise tests by using a portable patient monitoring system (IntelliVue MX450; Philips, Germany). The speech utterance signals of the CTT and tMTT that were sampled at 44.1 kHz and recorded during the last minute of each 2-min bout of the exercise stages using a wireless microphone were saved into the Praat voice analysis computer software program. Next, data for the utterance rates were obtained from the Praat script calculation (de Jong and Wempe, 2009) while data for the estimated exercise intensity based on the CTT and the tMTT approaches were measured by determining the percentage of the CTT (%CTT) (Loose et al., 2012) and the percentage of the tMTT (%tMTT), respectively. The %tMTT = (tMTT_exercise_/tMTT_rest_) × 100, where tMTT_exercise_ is the successful alphabet sounds uttered within a single breath during various stages of exercise while tMTT_rest_ is the successful alphabet sounds uttered at rest before exercise.

### Statistical Analyses

The data set was examined for normality by using the Shapiro–Wilk test. Thus, the utterance rate and the estimated exercise intensity for each TT (i.e., %tMTT and %CTT) were compared using a repeated-measure analysis of variance (ANOVA) for the within-subjects variable to identify any differences in %CTT or %tMTT when participants underwent several stages of incremental cardiorespiratory exercise. Spearman’s correlation analyses were also performed between the %tMTT and the %HRR, RPE and between the %CTT and the %HRR, RPE measures to evaluate the associations of the variables. All statistical analyses were performed using SPSS Statistics for Windows, v17.0 (IBM), and a probability of *p* < 0.05 was used to determine statistical significance.

## Results

**FIGURE 3:**
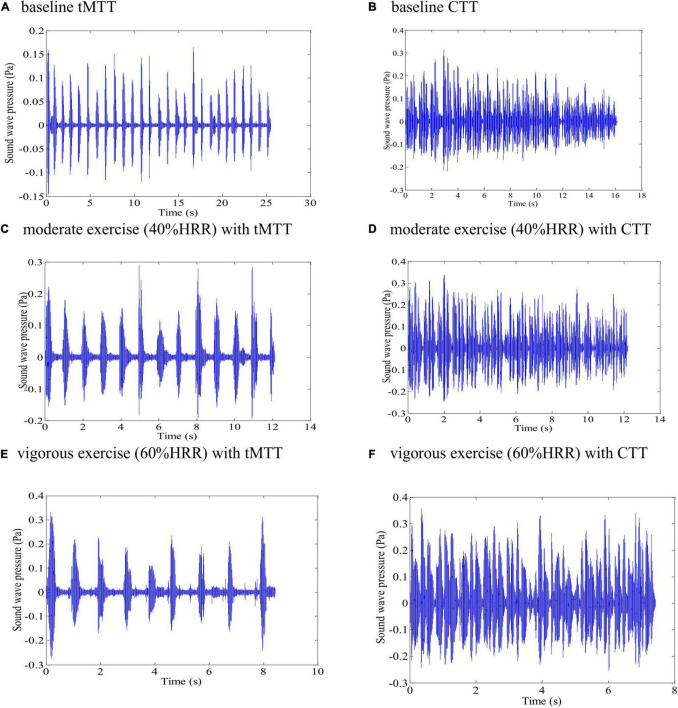
Different patterns of speech signals from one of the participants when producing tMTT versus CTT utterances over time within a single breath at various exercise stages: **(A)** baseline tMTT, **(B)** baseline CTT, **(C)** moderate exercise (40%HRR) with tMTT, **(D)** moderate exercise (40%HRR) with CTT, **(E)** vigorous exercise intensity (60% HRR) with tMTT, and **(F)** vigorous exercise intensity (60% HRR) with CTT.

**FIGURE 4:**
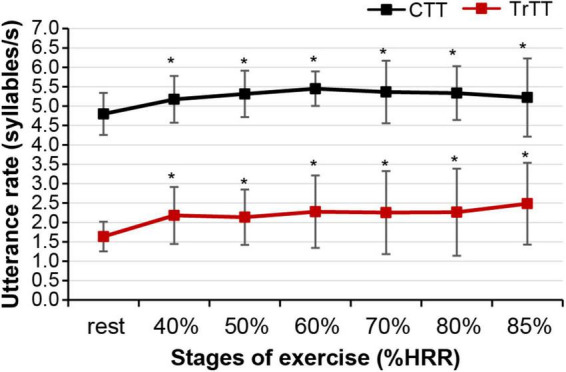
Mean utterance rates in the CTT and tMTT as represented by markers and standard deviation represented by error bars at the resting stage and incremental stages of exercise. Asterisks (*) denote significant differences in the utterance rate between the resting state and the respective stages of incremental exercise at a 0.05 significance level.

Figure 3 shows the different patterns of utterance speech signals over time between the CTT and the tMTT that were produced within a single breath by one of the participants at baseline (rest) and while exercising at moderate (40%HRR) and vigorous stages (60%HRR). Incremental cardiorespiratory exercise had a significant effect on the utterance rates in both CTT [*F*(3.2,72.5) = 5.796, *p* = 0.001] and tMTT [*F*(3.3,75.9) = 2.996, p = 0.001]. In both TTs, the utterance rate, which is determined by dividing the total number of syllables by the utterance duration including pauses (de Jong and Wempe, 2009), in all stages of the exercise demonstrated significant differences (*p* < 0.05) in comparison with the utterance rate at baseline (i.e., resting stage) (see Figure 4).

**FIGURE 5:**
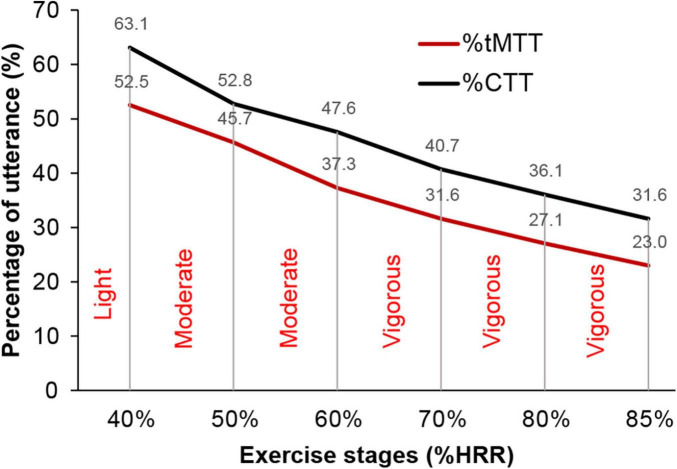
The negative gradient of relative scores (i.e.,%tMTT and %CTT) and exercise stages (%HRR). Intensities of exercise are categorized from light, moderate, and vigorous.

The mean CTT at rest (22 ± 4) indicates that the successful phrases participants can utter in a single breath sequentially at rest are until “twenty-two one thousand.” Meanwhile, the mean tMTT at rest was 32 ± 8, which denotes that the mean successful monosyllabic alphabets that participants could utter in a single breath sequentially at rest were until the letter/G/in the second round. Although the values at rest in both TTs were incomparable due to the differences in the syllables, the mean duration of utterance at rest for tMTT (31.8 ± 8.3 s) was significantly higher than that for the CTT (20.9 ± 4.4 s), which may indicate that the tMTT encourages participants to achieve further utterances within a single breath while at rest. Figure 5 shows that both the %tMTT and %CTT decrease as the exercise progresses from lower intensity to higher intensity, which may indicate that the greater anaerobic demand at higher exercise intensity caused more speech difficulty and thus decreased the achieved %tMTT and %CTT. Significant reductions in %CTT were observable at specific transitions of exercise stages, i.e., 36.9% reduction at 40% HRR (*p* < 0.001), 10.3% reduction at 50% HRR (*p* < 0.01), and 6.8% reduction at 70% HRR (*p* < 0.01) in comparison with the preceding stages of exercise. However, no significant changes in %CTT were found between 50 and 60% HRR, between 70 and 80% HRR, and between 80 and 85% HRR. These results have not been reported in previous studies on CTT. In contrast,%tMTT showed significant reductions in transitions to all stages of exercise, with a 47.5% reduction at 40% HRR (*p* < 0.001), 6.9% reduction at 50% HRR (*p* < 0.05), 8.4% reduction at 60% HRR (*p* < 0.05), 5.7% reduction at 70% HRR (*p* < 0.01), 4.5% reduction at 80% HRR (*p* < 0.01) and 4.1% reduction at 85% HRR (*p* < 0.001). Thus, the %tMTT reduction may signify whether participants were at the lower or upper-moderate level of exercise intensity or the lower, intermediate, or upper level of vigorous intensity.

**FIGURE 6:**
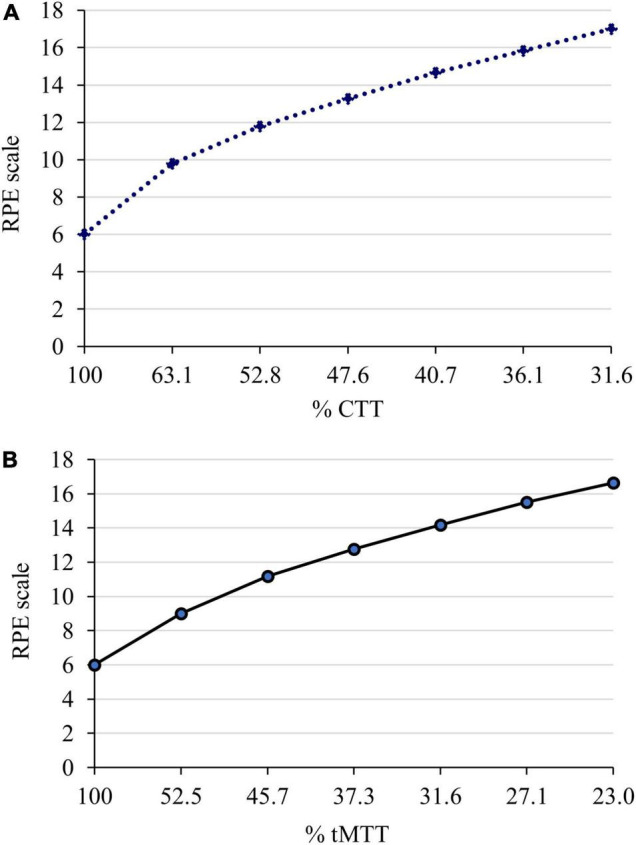
Serial response of perceived exertion based on Borg’s 6 to 20 RPE scale during two sessions of incremental cardiorespiratory exercise with **(A)** the CTT and **(B)** the tMTT, respectively. The estimated exercise intensity (%CTT and %tMTT) decreased as the exercise progressed. 100%CTT and 100%tMTT indicate the baseline where RPE scale is the lowest, 6 as there is no exertion.

Spearman’s correlation analyses were performed between the %tMTT and the %HRR and RPE and between the %CTT and the %HRR and RPE. Figure 6 illustrates the plots for these correlations, with a correlation coefficient (*r*_*s*_) of −0.53 for %tMTT and %HRR and −0.51 for %tMTT and RPE (*p* < 0.001 for both correlations). On the other hand, the correlation coefficient between %CTT and %HRR was −0.56 and that between %CTT and RPE was −0.45 (*p* < 0.001) for both correlations) (see Table 2). A previous study reported that the correlation coefficient was −0.93 for %CTT and %HRR and −0.86 for %CTT and RPE (Norman et al., 2008).

**TABLE 2:** Correlation coefficients (*r*_*s*_) between estimated exercise intensity based on respective percentages of tMTT and CTT against the %HRR and RPE.

Score	Exercise intensity measures		*p*-Values
	%HRR	RPE	
%tMTT	*r*_*s*_ = −0.53	*r*_*s*_ = −0.51	<0.001
%CTT	*r*_*s*_ = −0.56	*r*_*s*_ = −0.45	<0.001

*(d = 0.51).*

## Discussion

Our results show that only certain cardiorespiratory exercise stages could be distinguished in estimations by the CTT method, but the estimation of exercise intensity by the tMTT method could significantly distinguish all exercise stages. Since CTT may be preferred by therapists to estimate exercise intensity, this study highlights the importance of standardizing the TT protocol throughout the exercise test to ensure appropriate analysis and interpretation of the exercise intensity achieved by individuals since they considerably function as performance based-outcomes of exercise intensity using a relative measure against the baseline.

### Utterance Rate

In tMTT, all 25 letters were initially displayed in black color within a single frame inside the tablet. Each letter that needs to be uttered by the exercising individual changed its color from black to red, one after another at pre-set intervals of 1s (Figure 2). The display style and pre-set intervals were chosen due to their high repeatability for the utterance rate and the number of utterances at rest when tested in our preliminary study. Initially, we hypothesize that the utterance rates in the tMTT would be consistent from resting state (baseline) and across exercise stages as it is externally time-controlled while the utterance rates in the self-paced CTT would vary across exercise stages. However, we found that the utterance rates in the tMTT at any exercise stage were significantly different from the baseline utterance rate, but were more likely to be consistent across exercise stages. A similar pattern of findings was demonstrated during incremental exercises with the CTT. There are no significant differences in utterance rates in CTT across exercise stages but there was a significant difference between the baseline CTT and the first stage of exercise with CTT (Figure 4). It showed that maintaining utterance rates while exercising like in the baseline when there is no exertion, is a challenge regardless of using either the self-pace CTT or the externally controlled tMTT. The utterance rates in tMTT and CTT increase vastly from baseline toward the initial stage of the exercise, which may be attributable to the abrupt increase in ventilation during the initial stage of exercise (Burton et al., 2004; DiMenna and Jones, 2009) and the increased ventilatory oxygen demand for both speaking and exercise tasks (Dempsey et al., 2006; Baker et al., 2008). However, the rates are then maintained throughout exercise stages, probably due to the linear increase in ventilation with the work rate. Moreover, Bailey and Hoit (2002) reported that when both tasks are simultaneously performed, the challenge of coordinating speaking and breathing, especially in a high respiratory drive condition such as during progressive exercise, may interfere with the utterances, but such challenges may not be reflected in the present study as the utterance rates were more likely consistent at certain values for both TTs across stages of incremental exercise.

The timed-control approach in tMTT was initially hypothesized to enable the standardization of TT performance while exercising rather than allowing individuals to utter at their own’s pace and because timing is critical to individuals who engage in behavior to anticipate sensory events and prepare appropriate actions (Eagleman et al., 2005). However, the mean utterance rate of tMTT at a vigorous exercise intensity of 85% HRR was found to be as high as 2.5 syllables/s while in other stages of exercise, the utterance rates were maintained approximately at 2.0 syllables/s though the difference was not significant. On contrary, the mean utterance rates of CTT were consistent at approximately 5 syllables/s throughout the exercise stages. The higher utterance rate of tMTT observed at 85% HRR is more likely a response to speaking-related breathlessness and hypercapnic ventilatory drive to breath at higher exercise intensity (Hoit et al., 2007) that cause these participants to speak faster than they supposed to anticipate the time-controlled alphabets utterance in tMTT. Additionally, the anxiety of breathlessness at maximal exercise (Faull et al., 2016) could be a factor of the higher utterance rate of tMTT at the exercise stage of 85% HRR that drives the participants to cease talking while breath-holding and the urge to take another breath. Fortunately, the anxiety of breathlessness in those nearing maximal ventilation during intense exercise was not prominent in sedentary individuals (Faull et al., 2016) where participants in the present study were among the sedentary population.

### Exercise Intensity Estimation

An important finding of the present study is that the %tMTT significantly reduced from one stage of exercise to another in a gradual pattern. This is explained by the fact that ventilation increases linearly with work rates, but when speech and exercise are performed simultaneously, ventilation for speech production is reduced and speech becomes difficult (Meckel et al., 2002; Baker et al., 2008). This is because speech production is limited during the expiratory phases (Meckel et al., 2002; Creemers et al., 2017) and in the competitive ventilatory requirements created by simultaneous phonation and metabolic needs during exercise, non-phonated expirations predominantly occur to remove excess carbon dioxide for important metabolite functions (Bailey and Hoit, 2002), making the speaking task difficult and thus, reducing the utterance output produced.

On the other hand, the %CTT showed no significant changes between exercise stages of 50 and 60% HRR, between 70 and 80% HRR, and between 80 and 85% HRR. These results were not discussed in previous studies related to the CTT, where incremental exercise was set based on HRR progression (Norman et al., 2008; Loose et al., 2012), except in an earlier study by Norman et al. (2002) in which the %CTT was significantly different across exercise workloads corresponding to 50, 60, 75, and 85% of the HRR, which contradicted the findings observed in the present study. These contrasting results could be attributed to the differences in the formulas used for determining the age-predicted maximum heart rate in the HRR equation, wherein the present study equated age-predicted maximum heart rate to (208 − 0.7 × age) (Tanaka et al., 2001) while the previous study by Norman et al. (2002) equated it to a traditional formula (220 – age) before applying it into the HRR equation. The traditional equation for the age-predicted maximum heart rate was previously found to overestimate the maximal heart rates in young adults and increasingly underestimated the maximal heart rates as the participants’ age increased, before a revised equation of age-predicted maximum heart rate was introduced by Tanaka et al. (2001) and thus, adopted in the present study. Moreover, our findings suggested that the ability of the CTT to discriminate exercise intensities that are set based on targeted heat rates would probably be influenced by the approach used to determine the maximal heart rate such as a direct measure of maximal heart rate, the traditional equation of age-predicted maximal heart rate, or the revised equation of age-predicted maximal heart rate. Thus, the present study suggests that the tMTT approach could be more appropriately used to quantitatively distinguish the stage of incremental exercise.

Consistent with the study hypothesis, the %tMTT is significantly associated with HRR. The present study demonstrated that exercising at 23 to 53% tMTT would place the participants at a moderate (i.e., 40 to 59% HRR) to vigorous (i.e., 60 to 89% HRR) exercise intensity as described in ACSM’s Guidelines for Exercise Testing and Prescription (Pescatello et al., 2014). Moreover, 23% tMTT corresponded to 85% HRR while 53% tMTT corresponded to 40% HRR. Since this is the first study to examine the %tMTT during exercise, the findings obtained with the CTT were used as benchmarks. For a similar group of participants who performed the tMTT and CTT in two separate sessions, 41 to 63% CTT in the present study would correspond to a moderate to vigorous exercise intensity range, where 31% CTT corresponds to 85% HRR and 63% CTT corresponds to 40% HRR. This range of %CTT is higher than the range reported in a previous study (i.e., 33 to 50% CTT) (Norman et al., 2008) that used a similar exercise protocol in young adults. The young adults participating in that study were characterized by cardiorespiratory fitness at maximal oxygen uptake of 46.7 ± 8.1 mL/kg/min (Norman et al., 2008), which is approximately equivalent to 13.3 ± 2.3 METs, while the participants’ fitness in the present study was estimated to be 11.5 ± 2.2 METs (Table 1). The higher cardiorespiratory fitness seen in the previous study’s participants may not justify the lower range of %CTT values when compared to the present study because individuals with excellent aerobic fitness do not usually show dyspnea during exercise without any evidence of pathology. Instead, they are usually well accustomed to the ventilatory demands of exercise Smoliga et al. (2016), and thus may show greater utterance output with a higher %CTT than individuals with poor fitness. However, the cardiorespiratory fitness in the participants of the present study was limited due to estimation from a non-exercise test model with cross validity between 0.72 and 0.8 (Jurca et al., 2005). Meanwhile, the dissimilarity of the %CTT range values in the present study can be postulated to be attributed to the fact that participants in the present study were allowed to see a treadmill control panel that displays workload parameters such as distance, inclination, speed, heart rate, time, and calorie consumption, which were shielded from the participants’ view in the previous study (Norman et al., 2008). In the present study, the control panel was kept visible as usual for participants to view it if they wished to, but this was not essential because alterations in the visibility of conscious distance monitoring did not affect the exercise performance (Pinheiro et al., 2011). However, to our knowledge, no previous studies have related the effects of conscious spatial or physiological monitoring on speech output while exercising. This may indicate that shielding the treadmill control panel for spatial and physiological monitoring information, in contrast to the conditions in a usual treadmill exercise, could hinder feedback and induce participants to consciously control their walking or running pace on the treadmill instead of performing the exercise as an automatic action, which could interfere with their exercise performance (Wulf and Prinz, 2001) and thereby affect the corresponding estimation of exercise intensity using %CTT.

### Ratings of Perceived Exertion

In line with the study hypothesis, the findings of the present study showed correlations of tMTT with HRR and RPE with medium effect sizes (Field, 2013). Similar effect sizes were observed for correlations between CTT and HRR and between CTT and RPE, while a previous study reported larger effect sizes for similar correlations (Norman et al., 2008). The differences in the effect sizes between the present study and the previous study might be due to the larger sample size in the previous study, which involved 40 participants (Norman et al., 2008). Moreover, the present study showed that when the participants exercised at 50 and 60% HRR (moderate and vigorous exercise intensity), their %CTT was 53 and 48%, respectively, with RPEs of 12 (fairly light) and 13 (somewhat hard), respectively. Likewise, for the same exercise intensity, the %tMTT of the respondents was 46 and 37%, respectively, with RPEs of 11 (fairly light) and 13, respectively and this range of RPE was a recommended exercise intensity for that less trained individuals (Scherr et al., 2013). However, the RPE should be cautiously evaluated as it could increase significantly if the individuals encounter breathing resistance such as face mask-wearing while exercising (Poon et al., 2021) that leads to their discomfort and ultimately affects their perceived exertion. Fortunately, participants in our study did not wear a mask while exercising and performing the TT in a ventilated room.

### Practical Implications and Study Limitations

This study highlights an important protocol consideration that, until now, has not been addressed in the literature. First, clinicians need to understand the effects of different TTs on the speech utterance output when these tests are used to estimate and prescribe exercise intensity. Clinicians should also be cognizant when implementing cardiorespiratory exercises to achieve the desired rehabilitation outcomes. For example, if clinicians aim to utilize the self-paced CTT, patients should be able to maintain the required fluency or utterance rate to allow counting of phrases in each bout of exercise and to obtain an accurate utterance output as an estimate of exercise intensity. Our data showed that different stages of incremental exercise will result in significant step-wise reductions in %tMTT, but these reductions are unlikely to appear in %CTT, which will show no significant changes in certain %HRR stages; thus, the exercise intensity may not be well-discriminated when gauged using the self-paced CTT. As a result, the intensity of incremental exercise estimated from %CTT may not correspond to the differences in HR and RPE values as a result of the individual’s physiological responses and self-perceived exertion. Additionally, our study only utilized a targeted heart rate based on the individual HRR for each exercise stage. Therefore, the type, length, and rate of utterances used in the TT while performing the incremental cardiorespiratory exercise could result in different physiological outcomes. Thus, clinicians should strive to regulate the utterance rate of the TT across exercise stages to accurately monitor and later prescribe cardiorespiratory exercise. Additionally, it is important to note that step-wise reductions in utterance output are likely to not be perceived at the same rate as step-wise increases in exercise intensity. Since both of these rationales were solely hypothetical and since this study is, to our knowledge, the first study to measure utterance rates during the tMTT and the CTT, future research should further investigate the use of tMTT during cardiorespiratory exercise monitoring. Furthermore, the tMTT correlation was done only on a relatively small sample size of 30 young adults initially, of which only 24 completed the two exercise sessions. However, considering the number of participants (*n* = 24) and the moderate effect size (*d* = 0.51) for bivariate correlation in the present study, the statistical power from the *post hoc* power analysis remains acceptable, power (1-β) = 0.84. Thus, the present study still had an 84% chance of yielding a p-value less than 5% (Faul et al., 2007). This statistical power of 84% also exceeds the generally considered minimum desirable value (i.e., 80%) (Araujo and Frøyland, 2007). Lastly, this study did not examine the effects of deep breathing before the TT on the utterance rate and estimated exercise intensity, and future studies should investigate if these differences in the depth of breaths before the TT translate into improved cardiorespiratory exercise performance. Although this study did not implement a longitudinal design, it is important to translate these acute findings into practice to build on the current body of exercise monitoring and prescription literature.

## Conclusion

Counting talk test is likely to be sufficient if the determination of different exercise intensity is not a priority, while if the purpose of exercise monitoring is to classify the individuals’ exercise intensity as either light, moderate, or vigorous, the tMTT could be more universally employed. However, due to its larger variability in utterance rates across exercise intensities, the tMTT approach may alter the speech breathing of exercising individuals. The conclusion is that future studies are needed.

## Data Availability Statement

The raw data supporting the conclusions of this article will be made available by the authors, without undue reservation.

## Ethics Statement

The studies involving human participants were reviewed and approved by Medical Research and Ethics Committee, Ministry of Health Malaysia. The patients/participants provided their written informed consent to participate in this study. Written informed consent was obtained from the individual(s) for the publication of any potentially identifiable images or data included in this article.

## Author Contributions

SM participated in all aspects of the present study, data collection, data analysis, and creating the manuscript. RA participated in the data collection. LN and ES participated in the data analysis and manuscript preparation. All authors contributed to the article and approved the submitted version.

## Conflict of Interest

The authors declare that the research was conducted in the absence of any commercial or financial relationships that could be construed as a potential conflict of interest.

## Publisher’s Note

All claims expressed in this article are solely those of the authors and do not necessarily represent those of their affiliated organizations, or those of the publisher, the editors and the reviewers. Any product that may be evaluated in this article, or claim that may be made by its manufacturer, is not guaranteed or endorsed by the publisher.
